# Anti-inflammatory activities of carbon monoxide-releasing molecules (CO-RMs) in the brain

**DOI:** 10.1186/2193-1801-4-S1-L41

**Published:** 2015-06-12

**Authors:** Roberto Motterlini, Roberta Foresti

**Affiliations:** INSERM, France

**Keywords:** HO-1, carbon monoxide, microglia

The transcription factor Nrf2 and its downstream target heme oxygenase-1 (HO-1) are ubiquitous protective systems against oxidative stress, inflammation and tissue injury. The products of HO-1 enzymatic activity, biliverdin and carbon monoxide (CO), possess interesting anti-oxidant and anti-inflammatory properties suggesting that exploitation of this pathway as a target for drug discovery may offer therapeutic avenues in a variety of disorders [1]. In this context we have developed carbon monoxide-releasing molecules (CO-RMs), a class of compounds that deliver CO in a controlled fashion and exert a variety of pharmacological effects [2]. Data will be presented showing that CO-RMs modulate neuroinflammation and neuroprotection in vitro and in vivo. In BV2 microglia cells challenged with thrombin and interferon gamma, CO-RMs reduced the production of inflammatory mediators both in normoxic and hypoxic conditions. Similarly, in a rat model of collagenase-induced intracerebral hemorrhage, CO-RMs modulated microglia activation and the production of TNF-α while partially protecting against brain damage [3]. The mechanism(s) by which CO liberated from CO-RMs exert protection remains elusive. However, our laboratory has recently produced findings in BV2 microglia pointing to mitochondria as a plausible target for the anti-inflammatory action of CO. Studies are also currently ongoing on the synthesis and characterization of novel hybrid molecules that potently activate Nrf2 and simultaneously release CO in order to maximize the anti-inflammatory effects of the HO-1 pathway [4].
